# Common Genetic Factors May Play a Role in the Relationships Between Body Composition, Adipokines, and Low-Back-Pain-Related Disability

**DOI:** 10.3390/biom14111426

**Published:** 2024-11-08

**Authors:** Nader Tarabeih, Alexander Kalinkovich, Shai Ashkenazi, Adel Shalata, Gregory Livshits

**Affiliations:** 1 Department of Morphological Sciences, Adelson School of Medicine, Ariel University, Ariel 40700, Israel; nader@ariel.ac.il (N.T.); shaias@ariel.ac.il (S.A.); 2 Department of Anatomy and Anthropology, Faculty of Medicine, Tel-Aviv University, Tel-Aviv 69978, Israel; alexander.kalinkovich@gmail.com; 3 The Simon Winter Institute for Human Genetics, Bnai Zion Medical Center, The Ruth and Bruce Rappaport Faculty of Medicine, Technion-Israel Institute of Technology, Haifa 32000, Israel; adel.shalata@gmail.com

**Keywords:** adipokines, body composition, genetics factors, heritability, variance decomposition analysis

## Abstract

In this study, we evaluated the contribution of the putative genetic factors into the established associations between selected circulating adipokine levels, body composition measurements, and low-back-pain-related disability scores (LBP_DS). A total of 1078 individuals from 98 nuclear families (with 1 to 11 siblings per family) were examined. A detailed self-report questionnaire was used to collect LBP disability data; body composition (fat, skeletal muscle mass, and extracellular water (ECW)) was assessed using the bioimpedance method; plasma levels of adipokines were measured by ELISA. Pedigree-based statistical analysis methods were used, including family-based variance component analysis (VCA) and principal phenotype analysis (PPA), to estimate the contribution of potential genetic and environmental factors. The VCA revealed a significant additive genetic component in LBP_DS and for the selected body composition phenotypes and adipokines. The study also revealed that both adipokines (GDF-15, chemerin, and follistatin) and body composition variables (BMI, fat mass/weight, waist circumference, and ECW) were genetically correlated with LBP_DS. Next, PPA generated two synthetic phenotypes: PP_CT_ (combining cytokines) and PP_BC_ (combining body composition variables). There was no significant correlation between the putative genetic factors underlying the created PPs. However, each of them displayed a significant genetic correlation with LBP_DS. These findings indicate that genetic factors that are assumingly common for several adipokine variations and several body composition measurements, respectively, presumably have a pleotropic genetic influence on the LBP_DS variation, independently from one another. This, in turn, suggests that the alleged genetic factors employing pleiotropic effects on LBP_DS have a complex and probably non-overlapping composition.

## 1. Introduction

Despite the high socioeconomic impact of low back pain (LBP) and extensive research over the years, its etiology and pathogenesis remain unclear [1,2]. The current consensus argues that inflammation and metabolic disorders such as obesity are involved in LBP pathogenic processes, in which adipokines, overproduced by adipocytes in inflamed adipose tissue, play a key role [3,4,5]. The excessive production of adipokines has been shown to contribute to chronic metabolic- and inflammation-related disorders, such as cardiovascular disease, atherosclerosis, type 2 diabetes mellitus, and musculoskeletal disorders, including LBP [6,7,8,9].

According to WHO statistics, “LBP is the single leading cause of disability worldwide” [10]. LBP-related disability strongly depends on age and sex. For example, a recent cross-sectional observational study of 1214 US participants showed that The Oswestry Disability Index (ODI) in this sample clearly increased with age, reaching the highest mean ODI in ages 70–79 and the lowest in the 18–29 age group, being consistently higher in females [11]. We have recently reported a similar age-associated trend for Roland–Morris Disability scores (RMDQ) in a large, ethnically homogeneous Arab sample [8]. In this sample, we also observed significant associations between LBP-related disability scores (LBP_DS) and plasma levels of several adipokines and body composition characteristics. In particular, plasma levels of growth and differentiation factor 15 (GDF-15), a member of the transforming growth factor-β (TGF-β) superfamily [12], were found to be, in particular, significantly associated with LBP_DS (*p* = 2.95 × 10^−8^) [8]. GDF-15 is involved in various inflammation-mediated conditions and metabolic diseases [13,14]. It is expressed in adipose tissue and secreted by adipocytes [15], suggesting its role as an adipokine. Plasma levels of other adipokines, namely, chemerin, leptin, adipsin, and follistatin, also displayed significant associations with LBP_DS in this population [8,16]. However, the underlying mechanisms of these associations, in particular, the possible role of genetic factors, are poorly understood. Moreover, the existing literature is sparse and contradictory and considered mostly the putative genetic factors affecting obesity in relation to LBP [17,18]. One of the studies provided evidence that a high BMI has causal associations with the risks of various dorsopathies [19]. It should also be mentioned that, with rare exceptions, most of the studies used the BMI as a measure of obesity. However, this is a composite measure that includes other components of body composition, such as muscle and skeleton mass. This situation prompted us to examine more accurately and more comprehensively whether and to what extent familial (genetic) factors influencing inter-individual variations of obesity, other characteristics of body composition (assessed by the bioimpedance method, BIA [20]), and selected adipokines affect the genetic susceptibility to LBP_DS. The positive results would also suggest conducting the molecular genetic study of the most significant associations to clarify the nature of the genetic factors involved.

Several previous studies, including our own, have demonstrated a significant genetic component underlying an inter-individual variation of LBP, with heritability estimates often >30% [17,21,22,23,24], suggesting an important genetic/familial influence on LBP. However, whether the putative genetic factors affecting LBP also contribute to the correlations of LBP_DS with the body composition and the adipokines remains *terra incognita.*

Thus, the primary objective of this study was to evaluate the contribution of the putative genetic factors to the significant correlations between the LBP_DS and body composition characteristics and circulating levels of selected adipokines. Additionally, we aimed to evaluate whether and to what extent there are common latent heritable phenotypes underlying variations in several inter-correlated body composition and selected adipokine levels, respectively.

To achieve the goals of the study, we focused on an ethnically and culturally homogeneous sample of 98 nuclear Arab families in Israel, previously extensively studied by us with respect to various circulating, mainly pro-inflammatory biomarkers, body composition characteristics, LBP severity, and associated disability [25,26,27,28].

## 2. Materials and Methods

### 2.1. Study Population Design and Ethics

This project was originally designed as a case–control, community-based, cross-sectional study. LBP-affected and non-affected individuals were members of one of 98 nuclear families, with 1 to 11 siblings per family. The families were selected via proband (<50 years of age), previously diagnosed with LBP by a physician, confirmed by an orthopedist, and had at least one first-degree relative diagnosed with a similar condition. The collected data encompassed 1078 individuals, with ages ranging from 18 to 80 years old (mean age 43.0 ± 13.8 years). Participants were recruited and assessed in outpatient clinics in the city of Sakhnin (Israel) from January 2015 to January 2022. They were all from the ethnically and culturally homogeneous population of Israeli Arabs, sharing cultural and socioeconomic status. This allowed us to both diminish the genetic heterogeneity of the sample and enrich the sample for familial LBP cases.

The participants provided complete medical history and consented to provide access to their medical records and completed detailed disability questionnaire. The inclusion criterion for the study group was an age of 18 to 80 years. The exclusion criteria were pregnancy, traumatic disorders, fracture or surgery in the spine within the past 2 years, and severe heart problems. Certified and experienced nurses assessed all participants. Demographic data, anthropometrics, body composition measurements, and blood samples (30 mL) were collected from all participants.

This research was approved by the IRB-Helsinki Committee (Number: 042/2013K, Date: 4 November 2013) of the Meir Medical Center, Kfar Saba, Israel, and the Ethics Committee of Tel Aviv University, Tel Aviv, Israel. Written informed consent was obtained from all participants before their inclusion.

### 2.2. Assessment of Roland–Morris Disability Scores (RMDQ)

We used validated Arabic version of the RMDQ, which is one of the most commonly used questionnaires designed to assess self-rated physical disability caused by LBP [29]. This assessment generates a functional impairment score. The method yields reliable measures of disability and has been used in several studies of LBP (e.g., [30,31]). The RMDQ includes 24 items related to behaviors which can be affected by LBP. Each item is qualified with the phrase “because of my back pain” to ensure that the problem is due to back pain [31]. The RMDQ score is calculated as a simple sum of the number of items endorsed. The scores range from 0–24, with higher scores indicating greater levels of disability. The original distribution of the disability scores in our sample deviated substantially from normality and, therefore, was log-transformed to diminish the extent of the deviation. This score was considered as an indicator of LBP-related disability (LBP_DS) in the present study and was examined as a quantitative continuous phenotype.

### 2.3. Demographic, Anthropometric, and Body Composition Assessment

Demographic, anthropometric, and body composition data have been collected from the study population and recently described in detail [25,28,32]. They included anthropometric measurements: height (cm), weight (kg), calculated body mass index (BMI) in kg/m^2^, and waist circumference (cm). Body composition parameters were assessed using bioimpedance analysis (BIA), a safe, reliable, accurate, and inexpensive method, as described [32,33,34]. BIA gives several body-composition-associated measures, of which we included the evaluation of fat mass (FM) and skeletal muscle mass (SMM) in kilograms, and extracellular water (ECW) in liters. ECW was chosen due to its fundamental physiological significance [35], in particular, because it may serve as indicator of adiposity and inflammation [36]. Body mass components were used as ratios to body weight, such as FM/WT and SMM/WT, as they are interrelated and dependent on body weight.

### 2.4. Measurement of Adipokine Plasma Levels

Venous blood samples were collected from all study individuals after an overnight fast. They were centrifuged for 15 min at 1800× *g* at 4 °C within one hour of collection. Plasma fractions were separated and stored in aliquots at −80 °C. Levels of adipokines were determined by ELISA using the DuoSet kits (R&D systems, Minneapolis, MN, USA) according to the manufacturer’s protocols. The detection limits were as follows: 7.8 pg/mL for GDF-15, 46.9 pg/mL for follistatin, 16.7 pg/mL for chemerin, 31.2 pg/mL for leptin, 62.5 µg/mL for adiponectin, and 375 µg/mL for adipsin. The intra- and inter-assay coefficients of variation were between 2.3 and 8.6%. Before statistical analysis, the original measurements of the adipokines deviating from the normal distribution assumptions were log-transformed.

### 2.5. Statistical Analysis

Statistical analysis of the data was conducted using Statistica 64 (TIBCO Software, Version 13.5), without emphasizing on familial composition of the samples. All the measurements in the sample were compared between sexes by parametric (*t*-test) and non-parametric (Kruskal–Wallis) test. Pairwise Pearson correlation coefficients between biochemical factors and body compositions with LBP-disability were calculated. All data were subjected to z-transformation prior analysis.

### 2.6. Statistical–Genetic Analysis

After identifying body composition measurements and adipokines significantly associated with the LBP_DS, we proceeded to the quantitative genetic analysis using the MAN statistical package (Version 2020) (https://www.tau.ac.il/~idak/MAN_Manual.pdf, accessed on 17 July 2024). First, we estimated the contribution of potential genetic factors to the variation of each the traits of interest, using family-based univariate variance decomposition analysis (FVCA). This method decomposes the total phenotypic variation into components attributable to additive genetic factors (V_AD_), common family environment (V_CE_), and the residual (V_RS_) component of the variance. Next, we conducted family-based bivariate variance decomposition analysis to evaluate the contribution of the putative genetic factors and shared familial environment factors to the significant phenotypic correlation/association observed between the LBP_DS and its covariates (body composition and adipokines). The theory underlying this method was repeatedly described elsewhere [24,37]. The MAN package utilizes hierarchical (nested) testing of statistical–genetic models, comparing them through likelihood ratio tests (LRTs) to select the best-fitting and most parsimonious model.

Since several body composition phenotypes and adipokines were significantly associated with the LBP_DS, we wondered whether there is a common latent heritable phenotype underlying body composition phenotypes and adipokines, respectively. We therefore implemented principal phenotype analysis (PPA), which is described in detail in the MAN package. Analogous to principal component analysis, this method constructs a new trait, principal phenotype (PP), as linear combinations of the initial traits subjected to the analysis. The method maximizes the proportion of the variance of the PP explained by the specific component of variation, particularly the additive genetic variance. Thus, as a result of conducting PPA on several measurements related to adiposity (specifically, BMI, ECW, FM/WT, and waist circumference), we generated a composite phenotype, PP_BC_. Similarly, using data on several cytokines (GDF-15, chemerin, and follistatin), we generated phenotype PP_CT_. The objective for the application of this method is to construct the synthetic latent variable underlying the additive genetic variation of the composite traits and, therefore, typically possessing higher additive genetic component of the phenotypic variance. Next, we explored the extent of the shared putative genetic factors (genetic correlation) underlying the phenotypic correlation between the PPs and LBP_DS.

## 3. Results

### 3.1. Associations of Adipokine Plasma Levels and Body Composition Parameters with LBP_DS

The basic descriptive statistics for each of the study variables and for women and men are separately summarized in Table S1 (Supplementary Material). There was no difference in age between men and women in the study population. However, there were significant gender differences in body composition and most of the circulating levels of the adipokines. Thus, men had higher levels of adipsin and GDF-15, while women had higher levels of leptin and adiponectin, with no differences for chemerin and follistatin. Women had a higher BMI and FM/WT, while men had a higher waist circumference, SMM/WT, and ECW. Body composition variables and plasma leptin and chemerin levels were significantly inter-correlated in both sexes (Table S2).

**Figure 1:**
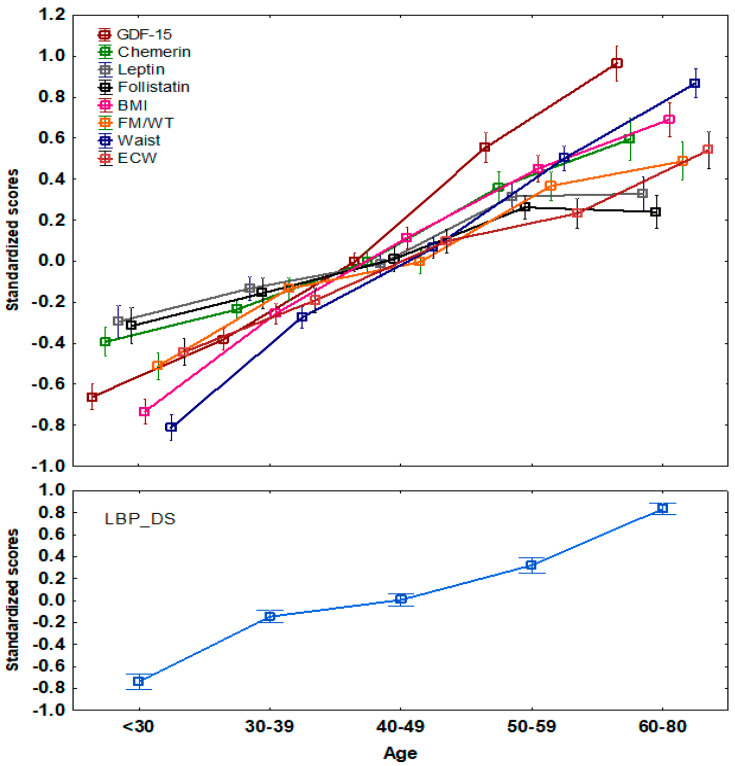
Plots of the age-related variations of the LBP_DS (**lower** panel) and selected body composition measurements and adipokines levels (**upper** panel). Data presented as mean ± standard error per age group and were subjected to z-transformation prior presentation.

The LBP_DS showed a clear age-dependent pattern (Figure 1). Parallel to this trend, the body composition measurements (BMI, waist circumference, FM/WT, and ECW) and plasma levels of the GDF-15, chemerin, and follistatin also increased with age. These correlations with age ranged between −0.65 and 0.64 and were statistically significant (*p* < 0.001) (Table S3, Supplementary Material). Noteworthy, all the correlations were best fit by a simple linear function. An important question that arises in this connection is whether the observed correlations between LBP_DS and the selected covariates (Figure 1, Table S3) are fully explained by age-dependent changes. Therefore, we further examined these correlations with the simultaneous adjustment for age and sex (Table 1). Except for the correlation of LBP_DS with SMM/WT and adiponectin, all other correlations survived adjustment and remained statistically significant, ranging from 0.171 to 0.402. These correlations were then subjected to quantitative genetic analysis.

### 3.2. Quantitative–Genetic Analysis

The parameter estimates and standard errors for the most parsimonious and best-fitting models with the results for both FVCA and bivariate FVCA are summarized in Table 2. As can be seen, the inter-individual variation of LBP_DS has a significant additive genetic component (V_AD_ = 0.2229 ± 0.0636). Additive genetic component estimates were statistically significant for all adipokines except adipsin, with GDF-15 showing the highest value (0.4289 ± 0.0899, *p* = 0.0001). All body composition measurements also demonstrated significant additive genetic components, with ECW achieving the highest value, V_AD_ = 0.5401 ± 0.0886.

Given the significant putative genetic component identified in variations of LPB_DS and several of its correlated adipokine and body composition variables, we conducted a series of bivariate variance component analyses. We aimed to determine whether the associations presented in Table 1 could be attributed to common genetic and/or environmental factors. Testing the correlations between the LBP_DS and adipokines, we found significant additive genetic correlations with GDF-15, chemerin, and follistatin levels, while leptin levels did not show a significant genetic correlation. Similarly, significant additive genetic correlations were observed for LBP_DS with the BMI, waist circumference, FM/WT, and ECW.

**Figure 2:**
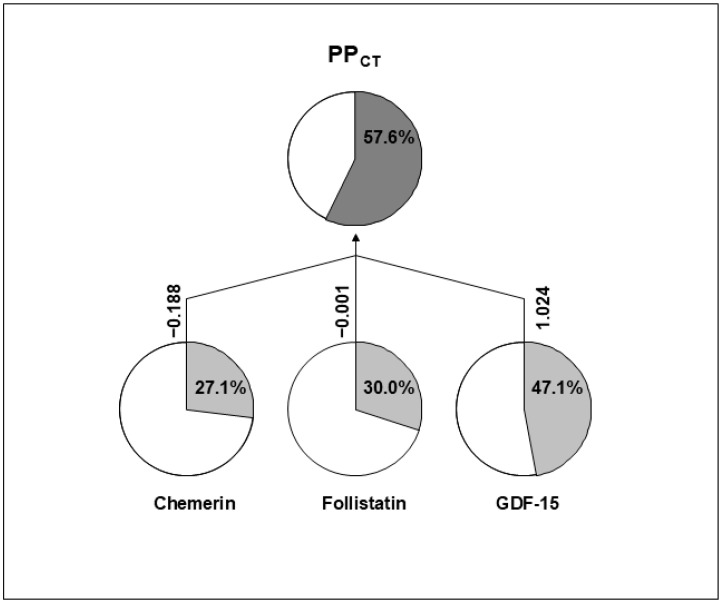
Diagram of composite phenotype (PP_CT_) generated using plasma levels of chemerin, follistatin, and GDF-15, through principal phenotypes analysis (PPA). The additive genetic variance in the percentage of PP_CT_ is explained by the additive genetic variance of each trait. Linear regression coefficients (β) for normalized traits are shown. The linear combination forms the PP_CT_-normalized trait with the maximal possible additive component.

**Figure 3:**
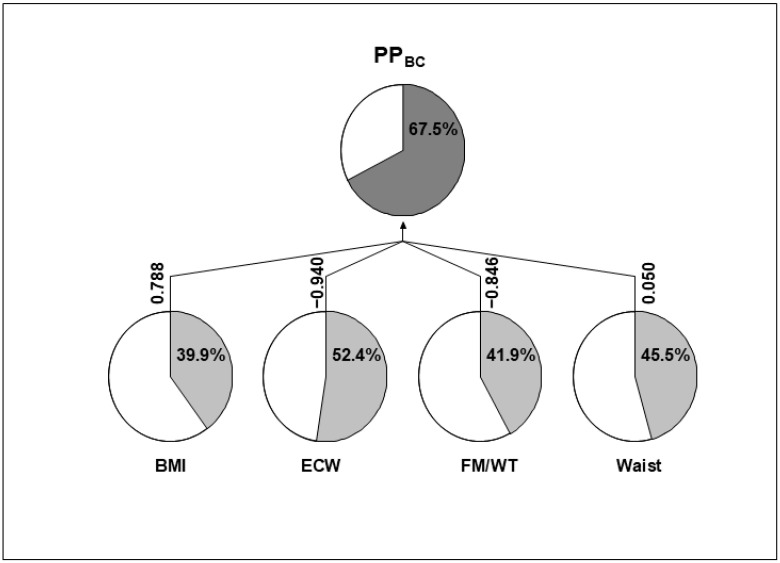
Diagram of composite phenotype (PP_BC_) generated using adiposity measurements, including BMI, ECW, FM/WT, and waist circumference, through principal phenotypes analysis (PPA). The PP_BC_ is explained in percentage by the additive genetic variance of each trait. Linear regression coefficients (β) for normalized traits are shown. The linear combination forms the PP_BC_ normalized trait with the maximum possible additive component.

Because variations in body composition and adipokine levels are not independent of each other, we applied a new approach called principal phenotype analysis (PPA). This method was implemented separately using the selected adipokines in one PPA and the selected body composition variables in another PPA. This resulted in two new composite traits: one combining the variations of the selected adipokines, and the second one combining the variations of the selected body composition variables. The first composite trait (PP_CT_) was derived from the analysis of GDF-15, chemerin, and follistatin (Figure 2). The second composite trait (PP_BC_) was derived from the analysis of BMI, waist circumference, FM/WT, and ECW (Figure 3). The additive genetic components of PP_CT_ were estimated to be 0.576 ± 0.107. Among the contributing adipokines, GDF-15 had the most substantial impact (regression coefficient β = 1.024), followed by chemerin with a negative effect (β = −0.188), while follistatin exerted only a minor contribution (β = −0.001). The additive genetic component of PP_BC_ was found to be 0.675 ± 0.116. BMI, ECW, and FM/WT were the primary contributors to this composite trait, while the waist circumference had a relatively minor effect.

We then examined the extent to which the generated PPs are correlated with each other, both phenotypically and genetically, implementing a family-based bivariate variance component analysis. The results of the analysis revealed no significant correlation; the accompanying *p*-values were >0.05. Next, we conducted a bivariate variance component analysis between each of the two composite PPs and LBP_DS to evaluate a possible common additive genetic effect (genetic correlations). The corresponding estimates in the most parsimonious and best-fitting models were 0.4372 ± 0.1054 with PP_CT_ and 0.2020 ± 0.0978 with PP_BC_.

## 4. Discussion

As mentioned in the Introduction, previous studies have suggested a significant genetic component underlying individual differences in LBP phenotypes. LBP_DS, associated with adipokines and body composition, remains largely unexplored, representing uncharted territory in the field.

The major research question of this study was whether the previously observed correlations between the LBP phenotypes and body composition, as well as some blood factors [8,16], have underlying familial (likely genetic) components. In the first stage of this research, we examined and confirmed several phenotypic correlations between the LBP_DS and selected covariates and showed that these correlations were significant and independent of age and sex. Our findings indicate a strong correlation between LBP_DS and markers of obesity, specifically the BMI, waist, FM/WT, and ECW (Table 1).

There is increasing evidence suggesting that changes in body composition are linked to LBP [38]. However, it is important to note that existing data on the potential relationship between body composition and LBP are still limited and contentious [39,40]. For instance, contrary to previous research, no significant links were observed between FM and LBP [40]. Observational and Mendelian randomization studies have indicated that a combination of factors may have a greater effect on LBP than any one factor alone. For example, the combined mediating effect of smoking and BMI is higher than BMI alone [41]. Additionally, our results indicate that the correlations between the LBP_DS and body composition parameters have underlying familial components. We found that these parameters have a significant additive genetic component (V_AD_ ranging between 0.3883 ± 0.0783 for BMI and 0.5401 ± 0.0886 for ECW). The involvement of genetic factors in the variation of body composition phenotypes, including fat and lean mass, has been reported previously [42,43]. However, the extent to which these factors can explain correlations between them and LBP_DS has not been sufficiently investigated. Thus, for the first time, our research offers insights into the genetic relationships between body composition measurements, such as the BMI, waist, FM/WT, and ECW with LBP_DS.

We found significant additive genetic correlations between LBP_DS and these indicators; they ranged from 0.3239 to 0.3924. Hussain et al. [44] reported that FM and fat distribution are associated with LBP intensity and disability, assuming that the systemic metabolic factors associated with adiposity play a major role in LBP pathogenesis. However, others have stated that adjusting for the possible effects of genetic and early shared environmental factors diminishes the significance of associations between obesity and LBP. For example, one study examined the effect of obesity-related measures on LBP when familial influences were and were not controlled [17]. The authors concluded that individuals who are obese or overweight are more likely to experience LBP than those in the normal weight range or those who are underweight. However, after controlling for familial factors, the association between obesity-related measures and LBP appeared to diminish substantially and was no longer evident after total adjustment. Thus, the existence of possible pleiotropic genetic effects is suggested, which was quantitatively assessed in the present analysis.

Adipose tissue is the major source of adipokines, which are often involved in and support inflammation associated with the pathogenesis of LBP. Our study clearly confirms this notion, as we observed several highly significant correlations between LBP_DS and adipokines. A quantitative genetic analysis of these correlations suggested an involvement of the putative genetic effects in the nature of LBP_DS correlations with the circulating levels of GDF-15, chemerin, and follistatin. The genetic correlation of LBP_DS with GDF-15 was, in particular, substantial (0.4296 ± 0.1049, *p* = 0.00006), and, therefore, represents a special interest. GDF-15 is a member of the transforming growth factor-beta (TGF-β) superfamily [12], known for its involvement in inflammation, metabolic regulation, and tissue repair [45,46]. In recent years, GDF-15 has garnered significant interest due to its association with various chronic conditions, including cardiovascular disease, obesity, and, more recently, musculoskeletal disorders like LBP-related disability [8,47]. Previous research has shown that genetic polymorphisms in the GDF-15 gene (e.g., SNPs in the promoter region) can influence its expression levels [14,48,49]. These genetic variations may modulate the role of GDF-15 levels in inflammatory responses and metabolic disturbances, which are critical in LBP pathogenesis and, therefore, require further genetic-molecular investigation.

Chemerin, a multifunctional protein, plays a considerable role in immune response and metabolism and is involved in inflammation and insulin resistance [50,51]. Our study shows its moderate but still significant genetic correlation with LBP_DS (0.2876 ± 0.1421). Genetic research has identified several variations in the gene that encodes chemerin, which affects its circulating levels and activity [52,53,54]. For example, variants in the RARRES2 gene are associated with serum chemerin levels and increase the risk of diabetic kidney disease in type 2 diabetes mellitus [52].

Additionally, our results show a significant genetic correlation of follistatin levels with LBP_DS (0.4055 ± 0.1566, *p* = 0.01). Follistatin, a glycoprotein known to antagonize the actions of several members of the TGFβ family, such as myostatin and activin A [55], and contribute to metabolic disorders, has also been shown to promote skeletal muscle hypertrophy [56], and affect cartilage formation, postnatal bone metabolism, and fracture healing [57,58]. A recent analysis revealed that the follistatin (FST) rs629725 A allele poses a significantly modest increased risk for acne vulgaris presentation [59]. The ability of follistatin to modulate inflammatory responses [60] and muscle growth [56] suggests that genetic variations affecting its levels may influence LBP_DS by influencing muscle integrity and inflammatory responses.

While the association of variations in specific body composition measures and adipokine levels with LBP_DS is certainly of great interest and importance, another question arises in this context: is there a common latent heritable phenotype underlying the body composition phenotypes and adipokine levels that is responsible for the observed correlations? PPA provides one approach to test this assumption. In this study, two synthetic PPs were generated and showed the non-equal contribution of the constituent phenotypes in each of them, PP_CT_ and PP_BC_. However, while GDF-15 clearly made a major contribution to PP_CT_, the BMI, ECW, and FM/WT contributed almost equally into PP_BC_, and the waist circumference exerted almost negligible contribution. There was no significant correlation between the putative genetic factors underlying the created PPs. However, each of them displayed a significant genetic correlation with LBP_DS (0.4372 ± 0.1054 with PP_CT_ and 0.2020 ± 0.0978 with PP_BC_). These findings indicate that genetic factors that are assumingly common for several adipokine variations and several body composition parameters are presumably exerting a pleiotropic genetic influence on LBP_DS variation independently of each other. This, in turn, suggests that the alleged genetic factors employing pleiotropic effects on LBP_DS have a complex and partly unrelated.

The cross-sectional design of this study has limitations as it does not allow for drawing conclusions about cause-and-effect relationships. Longitudinal studies are necessary in order to establish causality. An additional potential limitation of this study is related to the fact that it did not consider some other important and potentially relevant factors that may affect LBP_DS. These include metabolic diseases of bone (e.g., osteoporosis of the lumbar vertebrae), diabetes mellitus, and others.

On the other hand, this is a population-based study, with a low prevalence of the metabolic conditions, and has important advantages, including an ethnically and culturally homogeneous population, which likely reduce the influence of genetic and environmental covariates on sample variability.

## 5. Conclusions

In our study, we identified significant genetic correlations between the body composition parameters and adipokines with LBP_DS, thus providing valuable insights into LBP management and treatment. We believe that these findings may serve as a basis for further and more in-depth studies of the specific genetic mechanisms underlying the still poorly understood etiology and pathogenesis of LBP in particular. and other musculoskeletal disorders in general.

## Figures and Tables

**Table 1:** Correlation of LBP_DS scores with body composition parameters and plasma levels of adipokines.

Covariate	R	*p*	*p* ^a^
Age	0.481	<0.00001	-
BMI	0.280	<0.00001	0.04
FM/WT	0.227	<0.00001	0.03
SMM/WT	−0.230	<0.00001	NS
Waist	0.360	<0.00001	<0.00001
ECW	0.260	<0.00001	0.005
Adiponectin	0.031	NS	NS
Adipsin	0.224	<0.0001	0.0002
Chemerin	0.280	<0.00001	0.0002
Follistatin	0.171	<0.00001	0.006
GDF-15	0.402	<0.00001	<0.00001
Leptin	0.181	<0.00001	0.01

Pearson correlations (R) were computed for quantitative covariates. Correlation and corresponding *p*-values for all tests are shown prior and after adjustment (a) for age and sex. Abbreviations: LBP_DS, LBP-related disability; BMI, body mass index; FM/WT, fat mass/weight ratio; SMM/WT, skeletal muscle mass/weight ratio; ECW, extracellular water; GDF-15, growth and differentiation factor 15; NS, non-significant.

**Table 2:** Summary of the series of variance component analyses of the studied phenotypes. The additive genetic components estimated in most parsimonious models, along with bivariate variance component analysis of the levels of adipokines and body composition with the LBP_DS in the study sample, are shown.

Variable	Additive Genetic Variance (V_AD_)		Additive Genetic Correlation (R_AD_) with LBP_DS	
LBP_DS	0.2229 ± 0.0636	*p* = 0.000006	-	
BMI	0.3883 ± 0.0783	*p* = 1.86 × 10^−12^	0.3239 ± 0.1346	*p* = 0.01
FM/WT	0.3879 ± 0.0981	*p* = 3.52 × 10^−5^	0.3924 ± 0.1295	*p* = 0.002
Waist	0.4328 ± 0.0835	*p* = 6.08 × 10^−13^	0.3704 ± 0.1265	*p* = 0.003
ECW	0.5401 ± 0.0886	*p* = 7.90 × 10^−11^	0.3817 ± 0.1050	*p* = 0.0001
Adipsin	0.1199 ± 0.0772	*p* > 0.05	N/A	
Chemerin	0.2615 ± 0.0814	*p* = 0.0006	0.2876 ± 0.1421	*p* = 0.03
Follistatin	0.2949 ± 0.0744	*p* = 2.03 × 10^−8^	0.4055 ± 0.1566	*p* = 0.01
GDF-15	0.4289 ± 0.0899	*p* = 0.0001	0.4296 ± 0.1049	*p* = 0.00006
Leptin	0.3694 ± 0.0752	*p* = 1.94 × 10^−13^	0.1581 ± 0.1462	*p* > 0.05

V_AD_—additive genetic variance; R_AD_—additive genetic correlation; LBP_DS, LBP-related disability; BMI, body mass index; FM/WT, fat mass/weight ratio; ECW, extracellular water; GDF-15, growth and differentiation factor 15; N/A—not applicable due to non-significant estimate of V_AD_. Before conducting the analysis, all variables were adjusted for age and sex.

## Data Availability

The data are contained within the article and Supplementary Material.

## Supplementary Materials

The following supporting information can be found at: https://www.mdpi.com/article/10.3390/biom14111426/s1: Table S1: Descriptive statistics for studied biochemical markers and body composition variables. The data are presented for men (M) and women (F) separately. The gender differences (not adjusted for age) compared by the Mann–Whitney U test (*p*-values are shown); Table S2: Pearson correlations between body composition parameters and plasma levels of adipokines in the study sample, in men and women separately; male and female correlations are shown above and below the diagonal, respectively. All variables were adjusted for age before the analysis; Table S3: Correlations (R) between plasma levels of adipokines and body composition parameters with age for men (M) and women (F) separately.
