# Supplementary material for: Common Genetic Factors May Play a Role in the Relationships Between Body Composition, Adipokines, and Low-Back-Pain-Related Disability
Source: Biomolecules. 2024 Nov 8;14(11):1426. doi: 10.3390/biom14111426 (PMC11591575; doi:10.3390/biom14111426)
Supplement: Supplementary file 1 [file biomolecules-14-01426-s001.zip › biomolecules-3252830-supplementary.pdf]

Table S1. Descriptive statistics for studied biochemical markers and body composition variables. The data are presented for men (M) and women (F) separately. The gender differences (not adjusted for age) compared by the Mann-Whitney U test (p-values are shown).

**Marker**	**Gender**	**N**	**Mean**	**Min**	**Max**	**SD**	**Skewness**	**Kurtosis**	**U test for sex**
Age	M	485	43.04	16.27	101.0	14.24	0.45	0.30	NS
	F	582	43.27	16.43	92.0	14.80	0.17	-0.45	
BMI	M	479	27.54	16.98	43.77	4.30	0.39	0.30	0.006
	F	582	28.70	15.81	49.10	6.02	0.47	0.02	
FM/WT	M	475	0.25	0.05	0.44	0.05	-0.20	0.42	0.001
	F	568	0.36	0.09	0.57	0.07	-0.33	0.40	
SMM/WT	M	472	0.37	0.26	0.57	0.04	0.60	0.76	0.001
	F	566	0.27	0.17	0.43	0.04	0.52	-0.009	
Wasit	M	476	96.21	65.0	131.0	11.28	0.20	0.07	0.002
	F	579	93.96	61.0	134.0	14.52	0.19	-0.40	
ECW	M	475	20.69	14.00	35.79	3.17	0.77	1.44	0.001
	F	554	16.61	10.70	29.40	2.53	0.83	2.01	
Adiponectin	M	449	3.36	0.30	9.12	1.32	0.96	1.44	2.01×10^{-28}
	F	527	4.46	1.00	10.72	1.70	0.82	0.93	
Adipsin	M	472	0.23	-0.40	1.42	0.24	0.87	2.15	0.03
	F	567	0.20	-0.80	1.39	0.28	0.52	1.67	
Chemerin	M	446	88.51	27.70	177.10	26.03	0.39	0.24	NS
	F	531	90.21	29.48	175.53	27.23	0.55	0.08	
Follistatin	M	419	6.17	3.89	7.81	0.70	-0.73	0.74	NS
	F	470	6.11	3.86	7.72	0.77	-0.59	0.06	
GDF-15	M	448	8.18	5.75	11.08	0.73	0.32	0.62	0.00001
	F	526	8.62	7.04	11.28	0.71	0.64	0.30	
Leptin	M	474	2.06	-1.51	4.41	0.94	-0.71	1.07	0.001
	F	567	3.26	0.21	4.95	0.72	-0.75	0.82	

N, sample size; BMI, body mass index; FM/WT, fat mass/weight ratio; SMM/WT, skeletal muscle mass/weight ratio; ECW, extracellular water; GDF-15, growth differentiation factor-15; NS, non-significant.

Table S2. Pearson correlations between body composition parameters and plasma levels of adipokines in the study sample, in men and women separately; male and female correlations are shown above and below the diagonal, respectively All variables were adjusted for age before the analysis.

	BMI	Waist	FM/WT	SMM/WT	ECW	GDF-15	Chemerin	Adiponectin	Adipsin	Follistatin	Leptin
BMI		0.89	0.77	-0.75	0.60	NS	0.35	-0.12	0.15	0.11	0.62
Waist	0.82		0.50	-0.50	0.35	NS	0.23	-0.21	NS	NS	0.45
FM/WT	0.76	0.21		-0.97	0.43	0.12	0.30	-0.10	0.13	NS	0.68
SMM/WT	-0.66	-0.19	-0.93		-0.34	NS	-0.29	0.14	-0.10	NS	-0.69
ECW	0.52	0.10	0.53	-0.26		NS	0.23	NS	0.15	NS	0.35
GDF-15	0.12	0.12	0.13	-0.10	0.12		0.17	NS	0.16	NS	NS
Chemerin	0.27	0.10	0.29	-0.27	0.14	0.25		NS	0.17	0.16	0.37
Adiponectin	-0.17	-0.18	NS	NS	NS	NS	NS		0.10	NS	NS
Adipsin	0.18	NS	0.19	-0.12	0.21	0.15	0.14	0.14		NS	0.26
Follistatin	NS	NS	0.11	-0.10	NS	0.16	0.21	NS	NS		NS
Leptin	0.48	0.16	0.57	-0.55	0.27	0.14	0.37	NS	0.25	0.12	

BMI, body mass index; FM/WT, fat mass/weight ratio; SMM/WT, skeletal muscle mass/weight ratio; ECW, extracellular water; GDF-15, growth differentiation factor-15; NS, non-significant >0.05.

Table S3. Correlations (R) between plasma levels of adipokines and body composition parameters with age for men (M) and women (F) separately.

Marker	Gender	R	p-value
BMI	M	0.23	<0.001
	F	0.60	<0.001
FM/WT	M	0.27	<0.001
	F	0.52	<0.001
SMM/WT	M	-0.42	<0.001
	F	-0.65	<0.001
Waist	M	0.42	<0.001
	F	0.64	<0.001
ECW	M	0.29	<0.001
	F	0.49	<0.001
Adiponectin	M	0.03	0.205
	F	-0.01	0.655
Adipsin	M	0.20	<0.001
	F	0.33	<0.001
Chemerin	M	0.23	<0.001
	F	0.46	<0.001
Follistatin	M	0.11	0.04
	F	0.2	<0.001
GDF-15	M	0.53	<0.001
	F	0.59	<0.001
Leptin	M	0.20	<0.001
	F	0.35	<0.001

BMI, body mass index; FM/WT, fat mass/weight ratio; SMM/WT, skeletal muscle mass/weight ratio; ECW, extracellular water; GDF-15, growth differentiation factor-15.
